# Resolution of the cellular proteome of the nucleocapsid protein from a highly pathogenic isolate of porcine reproductive and respiratory syndrome virus identifies PARP-1 as a cellular target whose interaction is critical for virus biology

**DOI:** 10.1016/j.vetmic.2014.11.023

**Published:** 2015-03-23

**Authors:** Long Liu, Zoe Lear, David J. Hughes, Weining Wu, En-min Zhou, Adrian Whitehouse, Hongying Chen, Julian A. Hiscox

**Affiliations:** a College of Life Sciences, Northwest A&F University, Yangling, China; b School of Molecular and Cellular Biology and Astbury Centre for Structural Molecular Biology, University of Leeds, Leeds, UK; c Department of Infection Biology, Institute of Infection and Global Health, University of Liverpool, Liverpool, UK; d College of Veterinary Medicine, Northwest A&F University, Yangling, China; e NIHR Health Protection Research Unit in Emerging and Zoonotic Infections, Liverpool, UK

**Keywords:** PRRSV, Nucleocapsid, PARP-1, Antivirus, 3-AB, Proteome

## Abstract

Porcine reproductive and respiratory syndrome virus (PRRSV) is a major threat to the swine industry and food security worldwide. The nucleocapsid (N) protein is a major structural protein of PRRSV. The primary function of this protein is to encapsidate the viral RNA genome, and it is also thought to participate in the modulation of host cell biology and recruitment of cellular factors to facilitate virus infection. In order to the better understand these latter roles the cellular interactome of PRRSV N protein was defined using label free quantitative proteomics. This identified several cellular factors that could interact with the N protein including poly [ADP-ribose] polymerase 1 (PARP-1), a cellular protein, which can add adenosine diphosphate ribose to a protein. Use of the PARP-1 small molecule inhibitor, 3-AB, in PRRSV infected cells demonstrated that PARP-1 was required and acted as an enhancer factor for virus biology. Serial growth of PRRSV in different concentrations of 3-AB did not yield viruses that were able to grow with wild type kinetics, suggesting that by targeting a cellular protein crucial for virus biology, resistant phenotypes did not emerge. This study provides further evidence that cellular proteins, which are critical for virus biology, can also be targeted to ablate virus growth and provide a high barrier for the emergence of drug resistance.

## Highlights

• Proteomics was used to identify the cellular interactome of PRRSV N protein.
• The interactome included translation factors and PARP-1.
• Inhibition of PARP-1 by the small molecule 3-AB resulted in a decrease in virus infection.
• Sustained treatment of PRRSV infected cells with 3-AB suggested resistance free antiviral activity.

## 1 Introduction

Porcine reproductive and respiratory syndrome virus (PRRSV) is a global threat to the swine industry and vaccination provides limited protection. During PRRSV infection one of the most abundant viral proteins produced within the cell is the nucleocapsid (N) protein. This protein has important roles in the virus life cycle and may have critical interactions with the host cell (Rowland et al., 1999; Sun et al., 2012). At a basic level N protein complexes with virus genomic RNA to form the ribonucleocapsid core and as such is an integral component of the enveloped virus particle (Song et al., 2011). Because the N protein forms a close association with genomic RNA and other virus proteins such as those involved in replication and components of the viral envelope, the protein has been postulate to play an important role in virus RNA synthesis and virion assembly, perhaps in the modulation of these events (Lee et al., 2006).

The interaction of N protein and the host cell has been supported by several studies showing the association of this viral protein with cellular proteins including those involved in nuclear import such as importin-α and importin-β, the nucleolar protein fibrillarin (Yoo et al., 2003), and inhibitor of MyoD (a protein belonging to myogenic regulatory factors) family-a domain-containing protein (Song et al., 2009). Interactome analysis of the N protein using over expression and EGFP-trapping coupled to LC–MS/MS revealed further potential interactions including association with proteins involved in translation initiation and RNA post-transcriptional modification, including poly(A) binding protein (PABP), inducible PABP (iPABP), translation initiation factor 4E (eIF4E) and heterogeneous nuclear ribonucleoprotein A1 (hnRNPA1) (Jourdan et al., 2012a). Use of RNA silencing of PABP mRNA and ablation of the protein in PRRSV infected cells highlighted a positive role for this protein in infection and demonstrated that host cell proteins are critical for virus biology (Wang et al., 2012).

In order to generate a more comprehensive analysis of cellular proteins that could complex with PRRSV N protein we used an interactome approach in which N protein was expressed and purified exogenously and used as bait. Whilst over-expression analysis coupled to EGFP traps can provide a useful way of determining the interactome of a particular viral (or cellular) protein (Jourdan et al., 2012a; Wu et al., 2012), over-expression can turn on cellular stress pathways and this may lead to a potential bias in the interactome. To identify potential cellular protein interactions with PRRSV N protein, recombinant expressed and purified protein was therefore used as bait and bound to nickel affinity beads and incubated with HEK293T cellular lysate. Label free proteomics was used to both identify and quantify proteins that associated with the N protein bait and the UBC9 control bait. One hundred and eight proteins were identified by two or more peptides bound to the N protein bait which were also four fold or more abundant than binding with the UBC9 control bait. Eighteen proteins were found to be common between this analysis and a previous analysis using EGFP-N expressed in cells as bait (Jourdan et al., 2012a), including PABPC4 and PABPC1 as the top hits.

One of the unique proteins identified in the current study was Poly [ADP-ribose] polymerase 1 (PARP-1), which although normally associated with DNA virus (e.g. (Grady et al., 2012; Ohsaki et al., 2004; Tempera et al., 2010)) and retrovirus biology (e.g. (Bueno et al., 2013)) has also been shown to be an enhancer factor for influenza virus biology (Bortz et al., 2011). Use of the PARP-1 inhibitor 3-aminobenzamide (3-AB) in PRRSV infected cells indicated that PARP-1 acted as a positive factor for virus biology, and sequential passage of the virus in the presence of 3-AB did not lead to breakthrough virus. This study also illustrated how determining the cellular interactome of a viral protein can be used to select small molecule inhibitors that can be used to target viral activity without the emergence of resistant breakthrough viruses.

## 2 Materials and methods

### 2.1 Expression of bait proteins

Single colonies from the pTriEx-PRRSV-N (in BL21(DE3)pLysS), pHisTEV30a-MBP (His-tagged maltose binding protein, in BL21(DE3)) and pHisTEV30a-Ubc9 (SUMO conjugating enzyme, in BL21(DE3)) were cultured overnight at 37 °C until stationary phase, diluted 1:20 in 100 ml and cultured further until mid-log phase was reached (ca. OD_600_ 0.5). Protein expression was induced by the addition of 0.4 mM isopropyl-β-d-thio-galactoside (IPTG). Uninduced samples served as negative controls. Bacteria were lysed in 5 ml lysis buffer (50 mM Tris (pH7.6), 300 mM NaCl, 10 mM imadaizole, 0.5% (v/v) Triton X-100, 1 mg/ml lysozyme, 10 μg/ml RNase A and 5 μg/ml DNaseI), sonicated and clarified by centrifugation. Proteins were bound to Ni-NTA beads (Qiagen) for 1 h at 4 °C, washed (50 mM Tris (pH 7.6), 300 mM NaCl, 1% (v/v) Triton X-100, 20 mM imadaizole) and their purity was checked by SDS–PAGE followed by Coomassie blue staining and immunoblotting using αHis antibodies.

### 2.2 His-tag interaction studies

Approximately 1 × 10^7^ HEK293T cells per interaction were washed in ice-cold PBS and resuspended in 1 ml of lysis buffer (50 mM Tris (pH 7.5), 150 mM NaCl, 1% (v/v)NP40,) with 1X protease inhibitor cocktail (Roche),incubated on ice for 15 min, and clarified by centrifugation at 16,000 × *g*, 4 °C. Cellular lysates were mixed with 20 μl Ni-NTA for 30 min in order to pre-clear the sample of any proteins which would non-specifically bind to the Ni-NTA matrix. Normalized Ni-NTA-bound protein (50 μl) was mixed with the HEK293T protein lysates overnight at 4 °C. The following day the beads-protein complexes were washed three times in ice-cold PBS and eluted in 50 μl 4X LDS sample buffer (Invitrogen) with 10% DTT analyzed by silver stain and Western blot and then analyzed by mass spectrophotometry.

### 2.3 LC–MS/MS

Protein samples generated by the pulldowns were separated by one-dimensional SDS–PAGE (4–12% bis-Tris Novex mini-gel, Invitrogen). The resulting separated proteins were cut from the gel in six slices and subjected to in-gel digestion with trypsin. Trypsin digested peptides were separated using an Ultimate U3000 nanoflow LC-system (Dionex Corporation) consisting of a solvent degasser, micro and nanoflow pumps, flow control module, UV detector and a thermostated autosampler. The HPLC system was coupled to a LTQ Orbitrap XL (Thermo Fisher Scientific Inc.) via a nano ES ion source (Proxeon Biosystems). Quantification was performed with MaxQuant version 1.0.7.4 (Cox and Mann, 2008).

### 2.4 Cells and virus

MARC-145 cells were cultured in Dulbecco's modified Eagle's medium (HyClone) containing 10% fetal bovine serum, 2 mM glutamine, 100 units/ml of penicillin and 100 units/ml of streptomycin, and maintained at 37 °C with 5% CO_2_. A highly pathogenic PRRSV strain, TA-12 (GenBank accession number HQ416720.1), was used for the virus infection studies. 3-Aminobenzamide (3-AB), a competitive inhibitor of PARP-1, was obtained from Sigma–Aldrich and dissolved in DMSO. 3-AB was obtained >99% pure as determined by TLC.

### 2.5 Cell viability assay

MARC-145 cells were seeded in 96-well cell culture plates at approximately 1 × 10^5^ cells/well and cultured for 20 h at 37 °C in 5% CO_2_. The medium was replaced with fresh DMEM supplemented with 2% FBS containing 0, 5, 10, 20, 40 mM 3-AB, then the cells were incubated for the indicated number of hours. Cell viability was analyzed by measuring the succinate dehydrogenase level with a MTT Cell Proliferation and Cytotoxicity Assay Kit (Beyotime, Beijing, PR China) according to the manufacturer's instruction. A dose–response curve was produced to show the effects of the drug on the cell viability.

### 2.6 3-AB treatment and virus infection

MARC-145 cells were infected with PRRSV at a MOI of 1. After 1 h absorption at 37 °C, virus inoculum was removed by washing with PBS and cells were maintained in DMEM supplemented with 2% FBS containing varying concentrations of 3-AB or the corresponding amount of dimethyl sulfoxide (DMSO, control) at 37 °C. Virus supernatant was harvested at intervals of 24 h and frozen at −80 °C until titration on Marc-145 cells which was done by endpoint dilution in 96 well plates.

### 2.7 Indirect immunofluorescence assay (IFA)

MARC-145 cells plated in 96-well plates were infected with PRRSV at a MOI of 1. At indicated time-points after infection, the infected cells were washed with PBS, fixed for 10 min with cold acetone/methanol (1/1 v/v) pre-chilled at −20 °C. After blocking with 5% milk, the fixed cells were incubated with swine anti-PRRSV serum for 1 h at 37 °C. Unbound antibody was washed three times with PBS containing 0.1% Tween-20 (PBST). FlTC-conjugated rabbit anti-pig IgG (Bioss, Beijing, PR China) was added and incubated for 1 h at 37 °C. After three washes with PBST, the cell observation was carried out with a fluorescence microscope (Olympus CKX41), and images were acquired using DP2-BSW software.

### 2.8 Western blotting assay

MARC-145 cells, plated in 12-well plates, were harvested after infection with PRRSV and treatment with 3-AB or DMSO at the indicated time points. Cell lysates were subjected to 10% sodium dodecyl sulfate–polyacrylamide gel electrophoresis (SDS–PAGE) followed by blotting onto polyvinylidene fluoride (PVDF) membranes (Millipore, Massachusetts, USA). The membranes were primarily probed with rabbit anti-PARP antibody (Beyotime), swine anti-PRRSV-1 serum (Liu et al., 2010), or mouse anti-β-actin mAb (CWBIO, Beijing, PR China). After washing three times with TBS containing 0.1% Tween-20 (TBST), the membranes were incubated with horseradish peroxidase-conjugated goat anti-rabbit IgG (CWBIO), goat anti-swine IgG (Earthox, San Francisco, USA) or goat anti-mouse IgG (CWBIO) at 37 °C for 2 h, respectively. Detection was performed using enhanced chemiluminescence (ECL) reagents (CWBIO) according to the manufacturer's instructions.

### 2.9 Quantitative RT-PCR

Total RNA was extracted from PRRSV infected Marc-145 cells at 24 hpi using Trizol Reagent (CWBIO, Beijing, PR China). Reverse transcription was completed using PrimScript 1st Strand cDNA Synthesis Kit (TaKaRa, Dalian, PR China) according to the manufacturer's instructions. Quantitative real time RT-PCR amplification was performed on CFX96 Real Time PCR System (BIO-RAD, USA) using SYBR Primix Ex Taq II (TaKaRa). The viral genomic RNA was detected using primers Nsp1-F (5′-CACCTTGCTTCCGGAGTTG-3′) and Nsp1-R (5′-GAGAGACCGTGCACTGAGACATC-3′), and viral subgenomic RNAs were detected using primers N-F (5′-CCCGGGTTGAAAAGCCTCGTGT-3′) and N-R (5′-GGCTTCTCCGGGTTTTTCTTCCTA-3′). The GAPDH mRNA was amplified as the internal control represented the cellular mRNA level, using primers GAPDH-F (5′-GCAAAGACTGAACCCACTAATTT-3′) and GAPDH-R (5′-TTGCCTCTGTTGTTACTTGGAGAT-3′). The reaction conditions were as follows: 95 °C for 30 s; 40 cycles of 95 °C for 5 s and 60 °C for 30 s. Relative levels of viral subgenomic and genomic RNA were calculated using the 2^−△△*T*^ method of relative quantification with GAPDH.

### 2.10 3-AB virus sensitivity assay

The 3-AB sensitivity assay was carried out in a similar method as that used to assess the effect of HSP90 inhibitors against HRSV (Geller et al., 2013). Marc-145 cells were infected with PRRSV strain TA-12 as described. Following infection, the media was replaced with maintenance media containing 0.625 mM 3-AB, and infection allowed to continue until CPE was observed. The resulting virus was then used to infect fresh Marc-145 cells and the 3-AB concentration was doubled. This procedure was repeated until the drug concentration reached 20 mM (passage 6), then the drug concentration was kept constant until 15 passages were completed. The sensitivity of the resulting virus at passage 15 was then assessed by endpoint dilution on Marc-145 cells, and compared to the original input virus before the treatment with 3-AB.

## 3 Results

To investigate the cellular interactions of the PRRSV N protein and to identify potential cellular proteins that played important function(s) in virus biology a label free proteomic approach was used. We have used a similar mass spectrometry approach to identify the cellular interactome of Zaire Ebola virus VP24 protein (Garcia-Dorival et al., 2014). This was coupled to interaction studies using recombinant His-tagged PRRSV N protein as bait to pull-down cellular interacting partners.

### 3.1 Pull-down of cellular interacting proteins

**Fig. 1:**
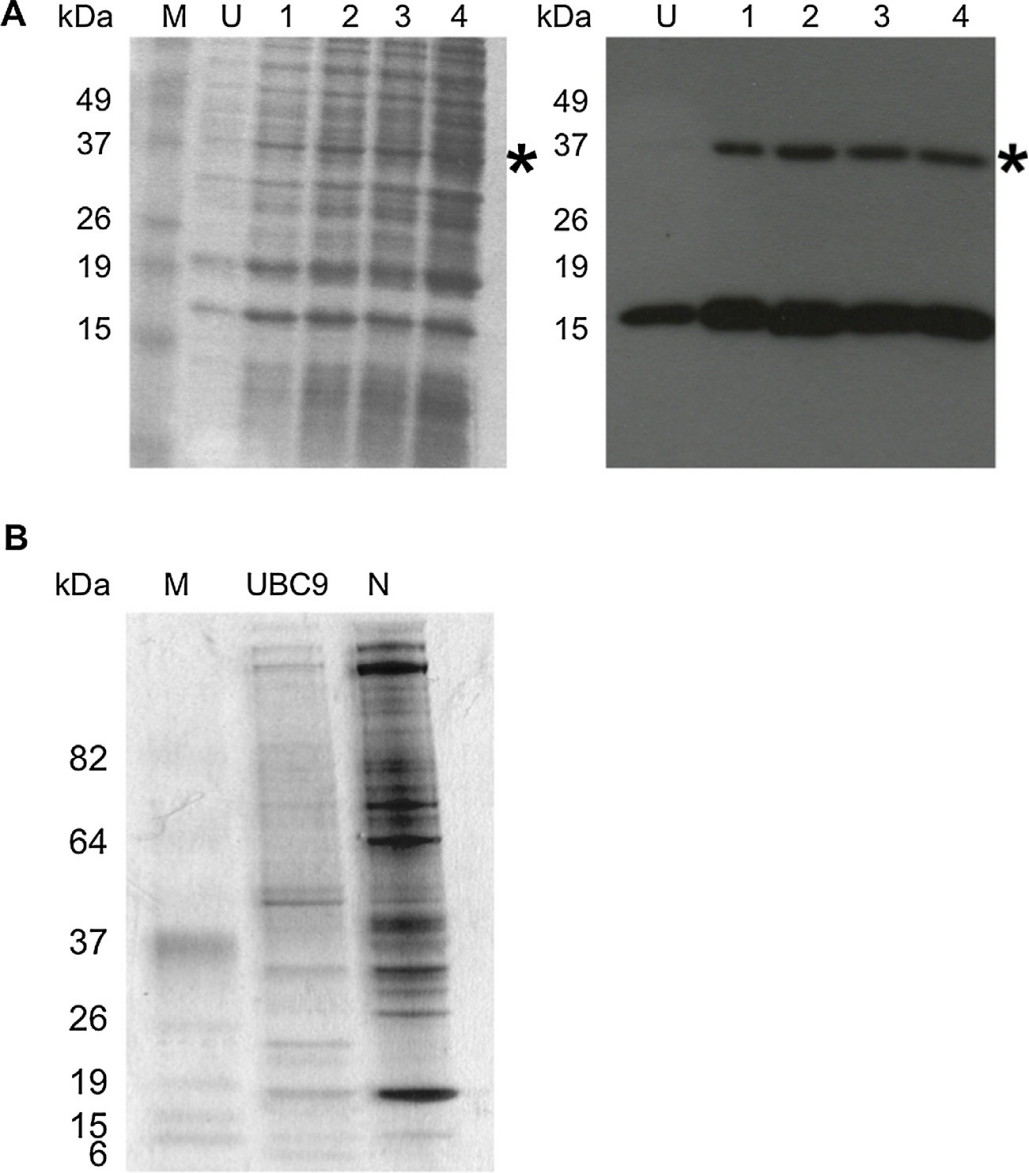
(A) Expression of recombinant His-tagged PRRSV-N protein in culture before (U) or after induction with IPTG at hourly times points (indicated). The position of marker proteins are indicated. N protein expression was confirmed by western blot using α-His antibody. The western blot showed His-specific antibody binding to monomeric N protein (∼15 kDa) and dimeric N protein (∼37 kDa), indicated by a *. (B) Silver stained gel of the 293 T cell lysate pulldown assay using either the UBC9 protein or PRRSV N protein as bait. Molecular weight markers are shown and indicated to the left.

N protein was expressed with a C-terminal His-tag (Jourdan et al., 2012b) in BL21(DE3)pLyS cells (Fig. 1A, left panel). To purify the N protein bacterial lysate from the induced culture was incubated with nickel affinity beads. Western blot analysis using anti-his antibody indicated that N protein bound to the nickel affinity beads (Fig. 1A, right panel). The same was also shown for the UBC9 protein used as a binding control (data not shown).

To identify potential cellular protein interactions with N protein, the nickel affinity bound N or UBC9 control protein (with similar molecular weight to N protein) were incubated with cell lystate prepared from HEK293T cells. This cell line was chosen because of the superior annotation of the human genome for protein identification and high level of transfection efficiency. Furthermore, numerous small molecule inhibitors have been identified for human proteins, which allowed us to test our hypothesis that proteomics can be a powerful tool for identifying therapeutic targets. Potential protein–protein interactions were visualized by silver stain analysis (Fig. 1B). The data indicated that cellular proteins were bound to both N and UBC9 and that these profiles were distinct from each other.

### 3.2 LC–MS/MS and bioinformatic analyses of the protein interactome

**Fig. 2:**
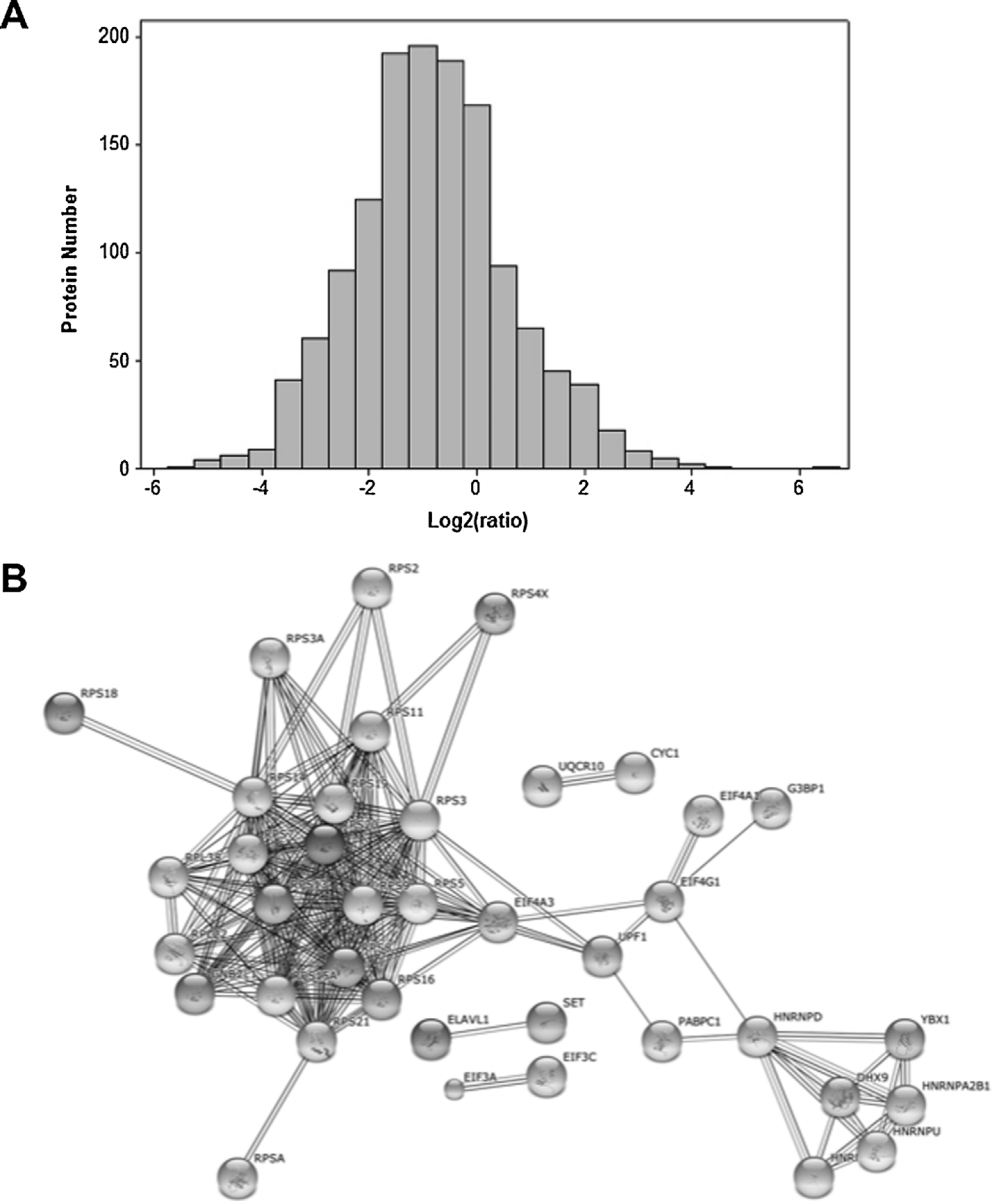
(A) Frequency of cellular proteins that interact with the PRRSV N protein as determined by ratio between the N protein target bait and UBC9 protein control bait. (B) Interactome map using the STRING algorithm of cellular proteins that bind to N protein, showing two major clusters, one of which clusters to ribosomal proteins (left).

Label free proteomics was used to compare the proteome isolated from the N interaction studies to the proteome isolated from the UBC9 pulldown. Peptide frequency was then used as a measure of the abundance of a cellular protein binding to the N protein bait compared to the UBC9 control protein. Confidence in protein identification is routinely achieved with the use of 2 or more peptides. Examination of this ratio for the N interactome dataset (Fig. 2A) indicated that the modal ratio was approximately Log_2_-1, and work by ourselves and others has previously suggested that the Log_2_-2 cutoff or more represents a working threshold for assigning specific from non-specific interactions that arise due to binding to either the matrix or the single chain EGFP antibody component of the GFP-trap (Trinkle-Mulcahy et al., 2008). Cellular interacting proteins exhibiting ratios of a conservative value of 4-fold or greater were therefore considered to potentially represent specific interacting partners of the N protein. This resulted in the identification of 108 proteins that could potentially interact with N protein either individually or as part of larger protein complexes (supplementary data). These proteins were used in downstream analysis.

Supplementary material related to this article can be found, in the online version, at http://dx.doi.org/10.1016/j.vetmic.2014.11.023.



STRING analysis was used to analyze the interactome data to identify cellular proteins that may act as larger functional complexes. These clustered into two main groups, one set involved in ribosome assembly and structure and the other set involved in nucleic acid binding and processing (Fig. 2B).

One hypothesis that could explain the dataset was that the degree of binding enrichment to N protein merely reflected the abundance of the specific protein in the total cellular proteome: i.e. the most abundant cellular proteins would be over-represented as binding with N protein. To investigate this we compared the N protein dataset with the PaxDb:Protein Abundance Across Organisms database (version 3.0) that ranks proteins according to their abundance in various human cell types based on spectral counting data. The eight most abundant proteins at the time of analysis were recorded as APOA2, RBP4, APOC2, TTR, ALB, APOA2, ORM1 and TMSB4X. However, none of these were part of the 108 cellular proteins associated with the N protein. There was no correlation between binding efficiency and protein abundance in the cell. For example, PABPC1 with the highest binding ratio of 85 is ranked 1079 out of 20480 proteins, whereas PARP-1 with a binding ratio of 13 is ranked 556 out of 20480 proteins. In contrast, TRIM26 is ranked 12950 out of 20480 and has a binding ratio of 18. Therefore, there was no apparent correlation between the abundance of a protein in a cell and its association with N protein.

### 3.3 Validation of N-interacting protein results by immunoblot analysis

**Fig. 3:**
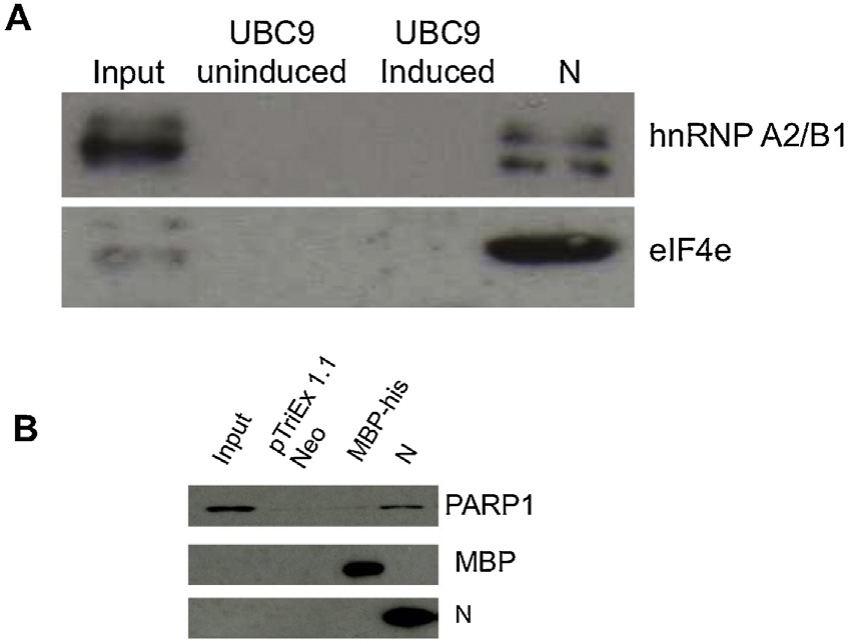
(A) Western blot validation of the interaction of two selected cellular proteins with N protein that were identified by LC–MS/MS, hnRNAP A2/B1 and eIF4e, shown are the input cell lysate from 293-T cells, uninduced and induced his-tag anchored UBC9 protein and his-tagged anchored N protein. (B) Western blot validation of the interaction of PARP-1 with the his-tagged PRRSV N protein used as bait. HEK293T cell lysates were incubated with control baits (his-tagged MBP and beads bound to bacterial lysates expressing empty pTriEx 1.1 Neo vector which was used to construct the N protein), or recombinant N protein bound to nickel beads. Cellular HEK293T lysate inputs are also shown in lane 1 from left. The cellular lysate inputs and pulldown outputs were detected by Western blot using antibodies against PARP-1 (upper panel), MBP (middle panel) or PRRSV N protein (bottom panel), respectively.

In our experience there is good agreement between using quantitative proteomics to identify interacting partners from pull down-based experiments and subsequent follow on experiments using specific analysis techniques (Emmott et al., 2013; Jourdan et al., 2012a; Wu et al., 2012). Nevertheless, aspects of the N protein interactome were validated by conducting repeat independent interaction studies of control and experimental systems and probing for specific virus and cellular proteins by western blot. We used two proteins with a lower and higher potential binding ratio; hnRNPA2/B1 (ratio 4) and eIF4 (ratio 45). Both of these proteins interacted with N protein but not UBC9 indicating the potential specificity of the interactions recorded by the LC–MS/MS analysis (Fig. 3A).

One of the unique proteins identified in the current study was Poly [ADP-ribose] polymerase 1 (PARP-1), which although normally associated with DNA virus (e.g. (Grady et al., 2012; Ohsaki et al., 2004; Tempera et al., 2010)) and retrovirus biology (e.g. (Bueno et al., 2013)) has also been shown to be a positive factor for influenza virus biology (Bortz et al., 2011). To confirm the interaction between PARP-1 and N protein, independent pulldown assays were performed. Recombinant protein from an empty negative control vector (pTriEx1.1 Neo), MBP-His control bait or recombinant N-His protein were purified using Nickel-affinity beads and incubated with a HEK293T cell lysate. Western blotting was then used to investigate whether PARP-1 was pulled down by the N protein compared to an MBP-His control bait (Fig. 3B). The data indicated that PARP-1 only associated with the N protein in contrast to the MBP-his control protein or the background matrix.

### 3.4 Inhibition of PARP-1 disrupts virus biology

**Fig. 4:**
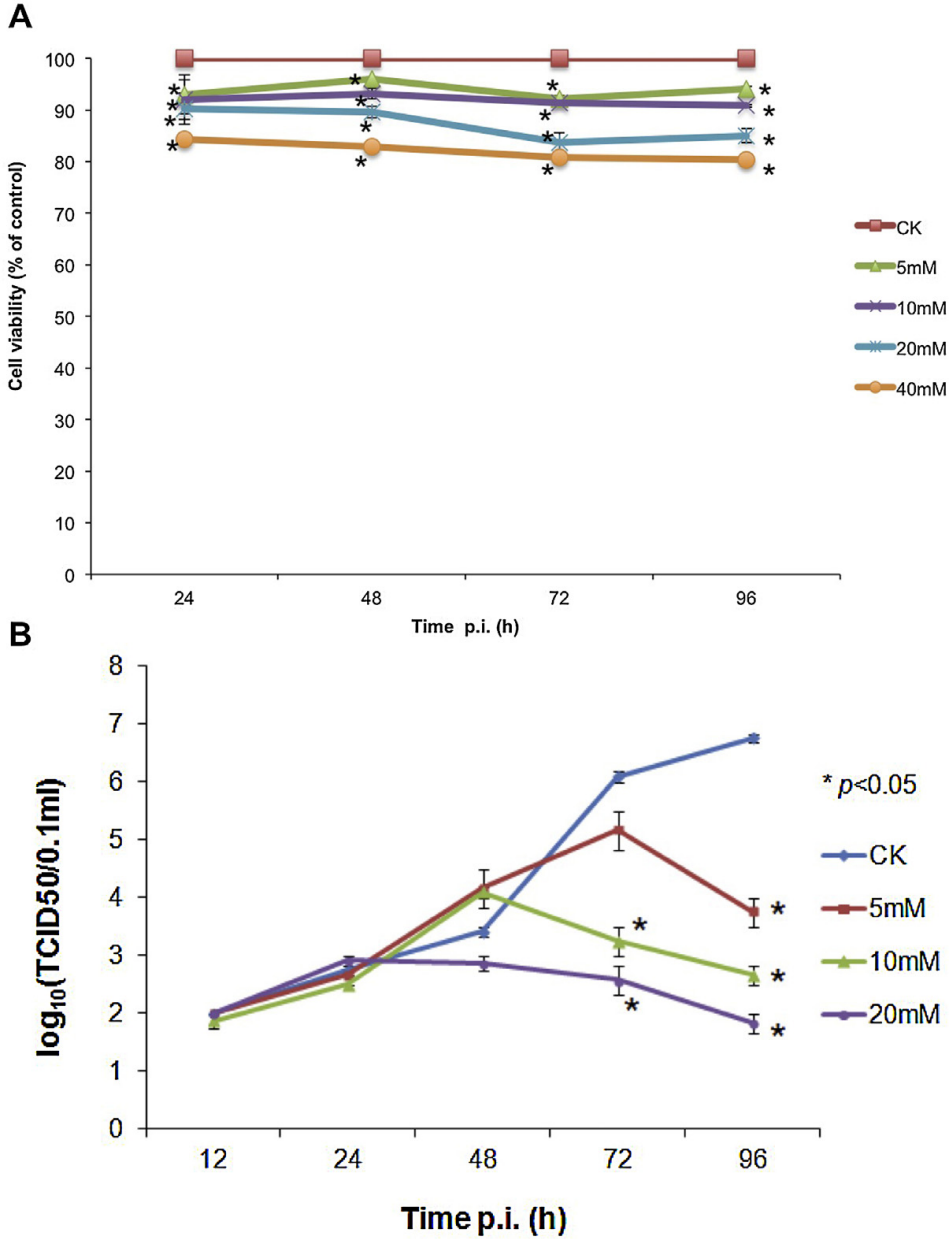
Inhibition of PARP-1 with 3-AB adversely affected the propagation of PRRSV in Marc-145. (A) Cell viability assay of Marc-145 cells cultured at various concentrations of 3-AB. Marc-145 cells were treated with the indicated concentrations of 3-AB and toxicity determined by measuring succinate dehydrogenase level at intervals of 24 h. One-way ANOVA test showed that 3-AB treated cells were less viable compared with control (*p* < 0.05). (B) Antiviral activity of PARP-1 in Marc-145. Cells were infected with PRRSV at MOI of 1 and maintained in the presence of indicated concentrations of 3-AB. Progeny viruses were harvested at intervals of 24 h and viral production was determined by endpoint dilution. One-way ANOVA test was done to compare 3-AB treated virus output with untreated control. The treatments resulted in significant differences were indicated with * (*p* < 0.05).

To investigate whether PARP-1 was important for virus biology, the small molecule inhibitor 3-aminobenzamide (3-AB) was used to treat PRRSV infected cells and virus biology assayed compared to control infected but untreated cells. First, several doses of 3-AB (5, 10, 20 and 40 mM) were assessed against untreated cells in a cell viability assay to determine the effect of the different doses over the virological assay period – 96 h. These doses were based on the reported activity of 3-AB against PARP-1 (Purnell and Whish, 1980). The data indicated that the doses had some effect on cell viability compared to the control untreated cells, ranging from approximately 80% viability after 96 h of treatment with 40 mM 3-AB to approximately 95% viability with 5 mM 3-AB (Fig. 4A). In most subsequent experiments 5, 10 and 20 mM 3-AB was used as this maintained sufficient levels of cell viability to assay virus biology over the complete 96 h experimental infection period.

**Fig. 5:**
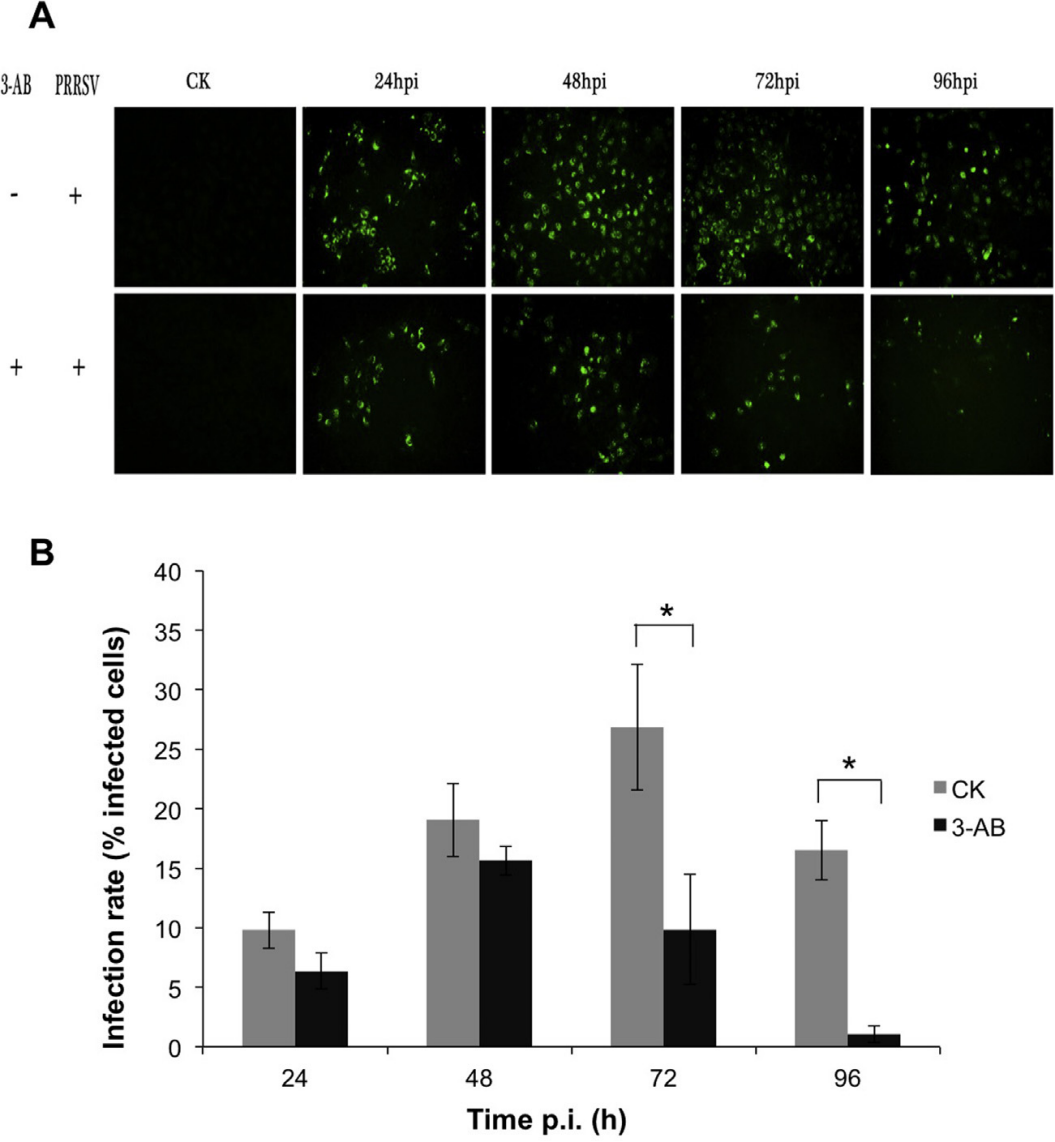
Immuno-fluorescence analysis of PRRSV infected Marc-145 with and without PARP inhibitor. Cells were incubated for indicated hours after infection and treatment with 20 mM 3-AB or DMSO as control. PRRSV infections were detected by IFA with swine anti-PRRSV serum and FlTC-conjugated rabbit anti-pig IgG antibody. (A) The immuno-fluorescence images were taken at an excitation wavelength of 495 nm with fluorescence microscope equipped with an appropriate filter. (B) Infection rate of Marc-145 determined by IFA. The percentage of infected cells were calculated by dividing the number of fluorescent cells by the total cells in the corresponding field. Each datum was obtained as the mean value from triplicate images.

Different doses of 3-AB were assessed for potential anti-viral activity by infecting cells with the same MOI of PRRSV and then treating cells with 5, 10, and 20 mM 3-AB over the infection period. Progeny virus production was compared by endpoint dilution assay to an untreated control at 12, 24, 48, 72 and 96 h post-infection. The data indicated that at the 12, 24 and 48 h time points, using a one-way ANOVA test, there was no significant difference (*p* > 0.05) in progeny virus production, however at 72 and 96 h post-infection there was a significant difference (*p* < 0.05) in progeny virus production between the treatments compared to the control non-treated infected cells (Fig. 4B). This was also confirmed using indirect immunofluorescence microscopy to detect viral antigens in virus-infected cells at 24, 48, 72 and 96 h post-infection compared to an untreated control (Fig. 5A). There were considerably less foci of infection in the treated cells compared to untreated control infected cells (Fig. 5B) (data shown for the 20 mM concentration of 3-AB). This confirmed the titration data that virus biology was adversely affected by 3-AB, with virus output being reduced to 1–2 log_10_ at 96 h post-infection compared to 7 log_10_ for the infected, but untreated control (Fig. 4B).

**Fig. 6:**
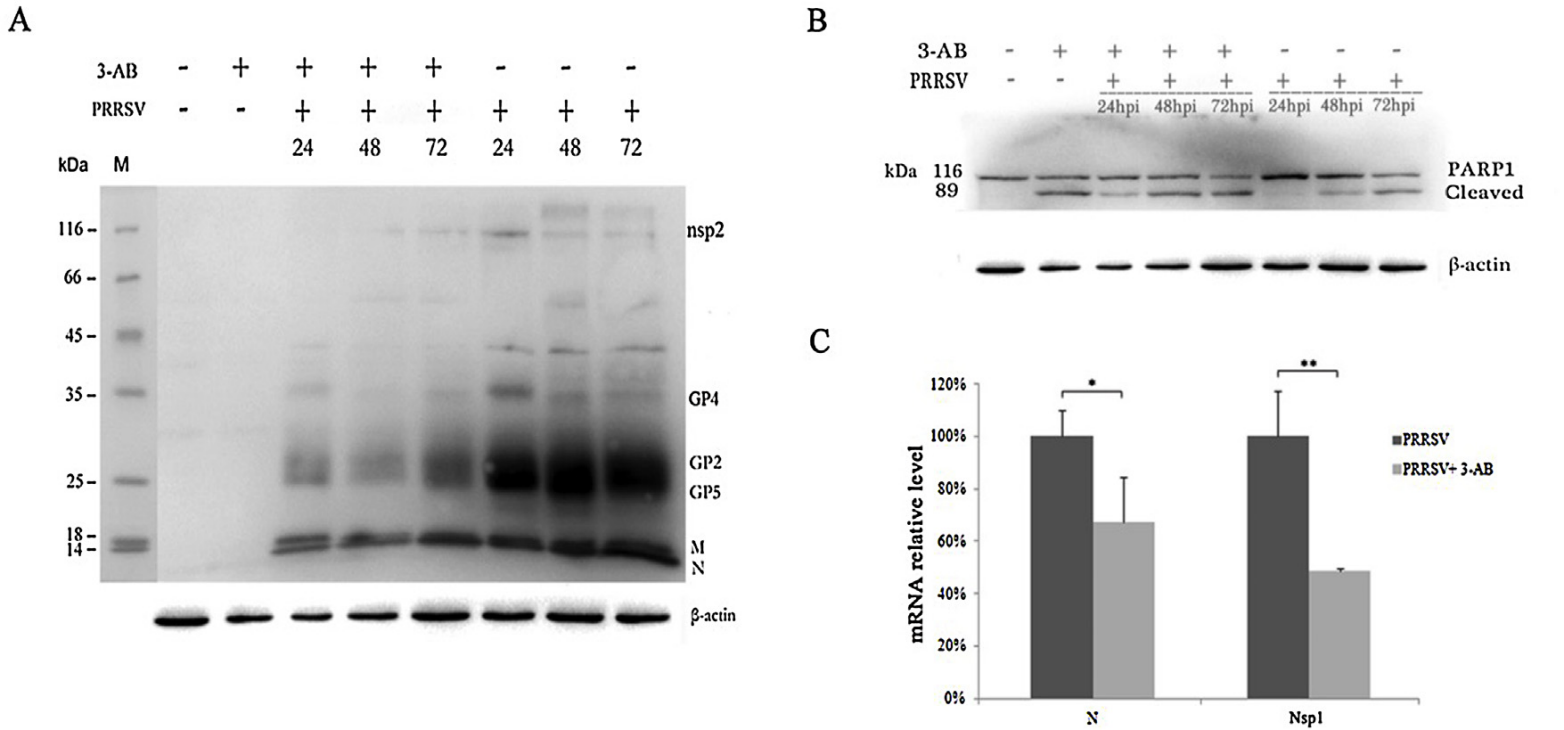
Validation of the antiviral effect of PARP-1 inhibitor in Marc-145. Cells were inoculated with 1 MOI of PRRSV and treated with 20 mM or without 3-AB. After incubation for the indicated time, cells were lysed and analyzed by Western blotting with swine anti-PRRSV antibody (A), and rabbit anti-PARP-1 antibody (B). Protein bands on the western blot were labeled according to previous reported studies (Li et al., 2012; Music and Gagnon, 2010). β-Actin was also detected by anti-β-actin antibody to normalize the protein amount of cell lysates loaded in lanes (lower panel in A & B). (C) Quantization of PRRSV genomic (Nsp1) and subgenomic (N) RNA in infected Marc-145 cells. Total RNA was extracted at 24hpi and subjected to real-time RT-PCR analysis. Relative levels of N and Nsp1 were calculated using GAPDH as an internal control. Data were shown as means ±SD of three independent experiments. One-way ANOVA test was done to compare N and Nsp1 RNA level in 3-AB treated cells with untreated control (*P < 0.05; **P < 0.01).

There are several stages to a virus life cycle, and the plaque assay provided a measure of the amount of virus being released into the supernatant. To investigate whether the PARP-1 inhibitor 3-AB affected virus biology in the cell, the abundance of viral proteins were compared using western blot at 24, 48 and 72 h post-infection between treated and untreated PRRSV-infected cells (Fig. 6A). The data indicated that at 24, 48 and 72 h post-infection nsp2, GP2, GP4, GP5, M and N proteins appeared less abundant in 3-AB treated cells than the non-treated cells (Fig. 6A). Western blot analysis indicated that in untreated and uninfected cells, PARP-1 was present in its native state. However, in cells treated with 20 mM 3-AB, PARP-1 is present as a cleavage product as well as native protein (Fig. 6B). This is consistent with the cell viability assay and that PARP-1 is cleaved during apoptosis. PARP-1 is present in the native and cleaved state in infected and treated cells at 24, 48 and 72 h post-infection. However, in untreated cells, PARP-1 is present in the native state at 24 h post-infection and in the native and cleaved state at 48 and 72 h post-infection. This is consistent with cleavage of PARP-1 previously reported in PRRSV infected cells (Lee and Kleiboeker, 2007).

As a protein responding to DNA damage or cellular stress, PARP-1 has been reported to be an enhancer factor for the optimal function of flu viral polymerase and knockdown of PARP-1 could result in reduced accumulation of NP in H5N1-infected A549 cells (Bortz et al., 2011). To investigate whether PARP-1 is involved in the replication and transcription of viral RNAs, viral genomic RNA (detected using primers in ORF1 encoding for Nsp1) and subgenomic RNAs (detected using primers in the gene encoding for N) in PRRSV infected cells were quantified by real time RT-PCR, and compared with the viral RNAs produced in PARP-1 inhibitor 3-AB treated cells. The result showed that inhibition the function of PARP-1 significantly reduced the production of PRRSV genomic (*P* < 0.01) and subgenomic RNAs (*P* < 0.05) (Fig. 6C), suggesting that PARP-1 acted as a positive factor for PRRSV infection.

### 3.5 3-AB exhibits resistance-free antiviral activity against PRRSV

**Fig. 7:**
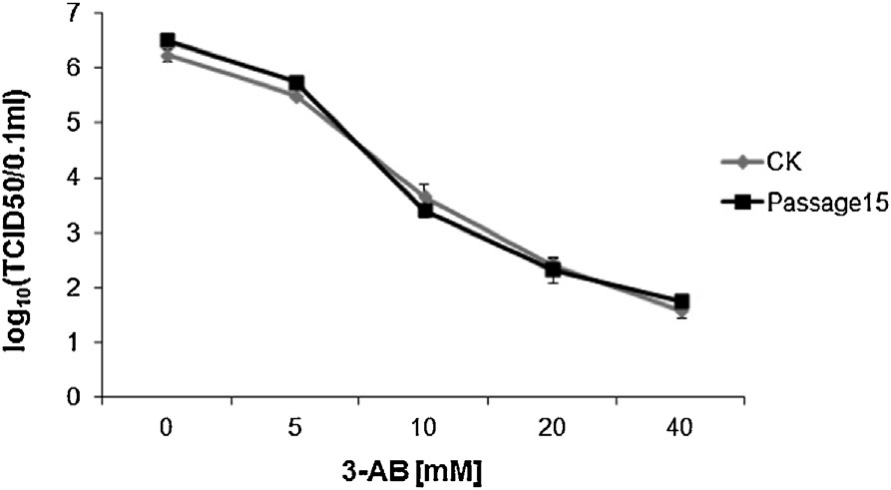
3-AB sensitivity assay based on an assay used to assess the effect of HSP90 inhibitors against HRSV (Geller et al., 2013). PRRSV was sequentially grown for 15 passages in various concentrations of 3-AB. Marc-145 cells were infected with PRRSV from the final passage or from the original stock for 72 h in the presence of indicated concentrations of 3-AB. Viruses released into the supernatant were titrated by endpoint dilution. Each datum represents the mean TCID50/0.1 ml of ≥2 experiments.

Viruses with RNA genomes can rapidly render antiviral compounds ineffective through acquisition of drug resistance by high mutation rates and this remains a major challenge in antiviral therapy. To investigate whether inhibiting PARP-1 with 3-AB maintained antiviral activity with sequential passage we adopted a similar approach to that used for screening HSP90 inhibitors against human respiratory syncytial virus (Geller et al., 2013). PRRSV was sequentially passaged 15 times in the presence of four different concentrations of 3-AB; 5, 10, 20 and 40 mM. Virus was taken at the end of passage 15 and its growth compared to the growth of virus that had not been passaged in the inhibitor and was exposed to the inhibitor for the first time. The passage 15 virus was equally as sensitive to 3-AB as the control virus (Fig. 7), indicating that prolonged treatment with the 3-AB PARP-1 inhibitor did not select for drug resistant viral mutants.

## 4 Discussion

RNA viruses such as PRRSV represent a major threat to global health and food security. Conventionally viral proteins have been targeted as part of anti-viral therapy, but due to the error prone nature of replication, resistant strains can rapidly emerge. Targeting cellular proteins that are critical for virus function may provide a therapeutic alternative, and increase the repertoire of anti-viral targets and therapies. Many cellular processes can be targeted transiently without long-term ill effects, and therefore such anti-cellular viral therapy may lend itself to acute rather than persistent infections. Targeting selected cellular proteins may provide broad-spectrum anti-viral activity. For example, inhibiting the function of protein chaperones (e.g. heat shock proteins) has been demonstrated for a wide range of viruses including infection of cells with influenza virus (Chase et al., 2008), HRSV (Geller et al., 2013; Radhakrishnan et al., 2010; Munday et al., 2014), rabies virus (Lahaye et al., 2012) and Ebola virus (Smith et al., 2010; Garcia-Dorival et al., 2014).

To identify cellular proteins that could be targeted as part of anti-viral strategies proteomics can be used. This can be at a global level to identify host cell metabolic processes (Dove et al., 2012; Emmott et al., 2010; Munday et al., 2010) or specifically identifying cellular proteins that interact with viral proteins (Emmott et al., 2013; Jourdan et al., 2012a; Wu et al., 2012). This study adopted the latter approach and used proteomics to determine the cellular interactome of PRRSV N protein. This interactome was elucidated under conditions that did not promote cellular stress pathways and involved the purification of a recombinant N protein as bait and pulldown assays used to identify cellular interacting partners from a cell lystate. Using a conservative selection criteria, 108 proteins were identified that could potentially interact with the N protein, or be part of larger complexes, which associated with the N protein. The selection criteria were based on cellular proteins being identified by two or more peptides and having a binding ratio higher than two fold compared to the control bait protein.

Analysis of the list identified PARP-1 as a cellular protein that could interact with the N protein and which also had a small molecule, 3-AB, that inhibited its function (Purnell and Whish, 1980). The inhibitor has been used both in vitro and in vivo. In the present study, the use of 3-AB demonstrated that PARP-1 activity was required for PRRSV biology, and therefore PARP-1 could be considered as positive viral factor. Our data have shown that PRRSV N protein interacts with PARP-1 and inhibition of PARP-1 significantly reduces the production of the viral genomic and subgenomic RNAs, indicating that PARP-1 may be recruited by N protein and benefit for the viral RNA replication and transcription processes. This result is consistent with the report that PARP-1 is a positive factor for the optimal function of viral polymerases in influenza infection, although the precise mechanisms remain to be resolved.

Prolonged treatment of PRRSV infected cells with 3-AB did not select for drug resistant viral mutants, at least over 15 passages. Here we used a similar strategy that was used to investigate an inhibitor of a cellular protein important for HRSV (Geller et al., 2013). Whilst we could have increased the concentration of 3-AB from 20 mM to 40 mM for the selection/reversion experiment, this latter concentration may have reduced the number of viral genomes by too much to generate sufficient numbers for selection experiments. Although it is hard to predict how many passages or viral generation times should be tested, it is worth noting that two amino acid substitutions in the N protein that abolish its nuclear localization (NLS) (Pei et al., 2008), underwent strong selection pressure both in vitro and in vivo that resulted in partial or complete reversion and gain of NLS activity (Pei et al., 2008).

Taken together our data suggests that that PARP-1 is important for the virus life cycle and cannot be substituted by the function of another cellular protein. In a wider context determining the cellular interactome of viral proteins can be used to identify cellular proteins whose functions can be ablated with small molecule inhibitors to disrupt virus biology. This adds to the general principle that is being established in anti-viral therapy (Baillie and Digard, 2013) that targeting cellular proteins may provide an attractive alternative and expand the repertoire of anti-viral inhibitors (Xiao et al., 2014; Munday et al., 2014; Garcia-Dorival et al., 2014).
