# Supplementary material for: Resolution of the cellular proteome of the nucleocapsid protein from a highly pathogenic isolate of porcine reproductive and respiratory syndrome virus identifies PARP-1 as a cellular target whose interaction is critical for virus biology
Source: Vet Microbiol. 2015 Mar 23;176(1-2):109–19. doi: 10.1016/j.vetmic.2014.11.023 (PMC4414928; doi:10.1016/j.vetmic.2014.11.023)
Supplement: Supplementary file 1 [file mmc1.docx]

Supplementary Data: Ratios of proteins identified from the PRRSV N protein immuno-precipitation experiment organised from potential strongest interaction to weakest interaction. Shown is the gene name of the protein, protein description, binding ratio (PRRSV N-his tag/UBC-his tag), number of different peptides used to identify the protein (peptide), sequence coverage and whether the protein has been identified in a previous study as interacting with the N protein. Proteins identified with one peptide and/or a binding ratio less than 4.00 have been excluded. Protein ID shows the International Protein Index (IPI) accession number. Ratio shows the (Heavy/Light) SILAC ratio. Peptides shows the number of unique peptides identified for each protein and SC (%) shows the sequence coverage for the protein this number represents. Posterior Error Probability (PEP) is a measure of the probability of misidentifying a protein

| Gene name | Protein description | Binding  Ratio | Peptide | Sequence coverage | Confirmation from previous study |
| --- | --- | --- | --- | --- | --- |
| PABPC1 | Polyadenylate-binding protein 1 | 85 | 20 | 33.02 | Jourdan et al. 2012.  PABP identified by Wang et al. 2012. |
| RPS3 | 40S ribosomal protein S3 | 85 | 10 | 54.32 |  |
| DHX9 | ATP-dependent RNA helicase A | 45 | 10 | 10.63 | Jourdan et al. 2012. |
| EIF4G1 | Eukaryotic translation initiation factor 4 gamma 1 | 45 | 6 | 7.73 |  |
| PABPC4 | Polyadenylate-binding protein 4 | 43 | 10 | 20.49 | Jourdan et al. 2012.  PABP identified by Wang et al. 2012. |
| GNB2L1 | Guanine nucleotide-binding protein subunit beta-2-like 1 | 41 | 11 | 47.32 | Jourdan et al. 2012. |
| PABPC3 | Polyadenylate-binding protein 3 | 38 | 10 | 17.91 | PABP identified by Wang et al. 2012. |
| RPSA | 40S ribosomal protein SA | 37 | 11 | 51.14 |  |
| IQGAP1 | Ras GTPase-activating-like protein IQGAP1 | 36 | 7 | 6.94 |  |
| RPS4X | Not assigned | 34 | 5 | 19.77 |  |
| RPS7 | 40S ribosomal protein S7 | 30 | 6 | 35.57 |  |
| RPS15A | 40S ribosomal protein S15a | 27 | 6 | 40.77 |  |
| NAP1L4 | Nucleosome assembly protein 1-like 4 | 27 | 5 | 43.93 |  |
| USP9X | Not assigned | 26 | 22 | 11.67 |  |
| HNRNPD | Heterogeneous nuclear ribonucleoprotein D0 | 25 | 4 | 36.61 |  |
| ANP32B | Acidic leucine-rich nuclear phosphoprotein 32 family member B | 25 | 4 | 22.31 |  |
| RPS8 | 40S ribosomal protein S8 | 24 | 6 | 40.38 |  |
| EMC1 | ER membrane protein complex subunit 1 | 24 | 12 | 20.64 |  |
| HNRNPU | Heterogeneous nuclear ribonucleoprotein U | 22 | 10 | 20.53 |  |
| RPS11 | 40S ribosomal protein S11 | 21 | 6 | 37.97 |  |
| NAP1L1 | Nucleosome assembly protein 1-like 1 | 21 | 7 | 23.50 |  |
| STT3B | Dolichyl-diphosphooligosaccharide--protein glycosyltransferase subunit STT3B | 21 | 4 | 5.45 |  |
| CYC1 | Cytochrome c1, heme protein, mitochondrial | 20 | 4 | 20.92 |  |
| SEC31A | Protein transport protein Sec31A | 20 | 13 | 15.44 |  |
| YBX1 | Nuclease-sensitive element-binding protein 1 | 19 | 4 | 30.86 | Jourdan et al. 2012. |
| EZR | Ezrin | 18 | 4 | 4.87 |  |
| CACYBP | Calcyclin-binding protein | 18 | 4 | 29.73 |  |
| TRIM26 | Tripartite motif-containing protein 26 | 18 | 3 | 10.02 |  |
| OSBPL9 | Oxysterol-binding protein-related protein 9 | 18 | 7 | 16.18 |  |
| RPL22 | 60S ribosomal protein L22-like 1 | 17 | 4 | 21.09 |  |
| LRRC59 | Leucine-rich repeat-containing protein 59 | 16 | 5 | 20.52 |  |
| RPS2 | Submitted name: RPS2 protein | 15 | 5 | 21.70 |  |
| USP24 | Ubiquitin carboxyl-terminal hydrolase 24 | 15 | 7 | 4.15 |  |
| USP9Y | Probable ubiquitin carboxyl-terminal hydrolase FAF-Y | 15 | 8 | 4.11 | Jourdan et al. 2012. |
| DHX30 | Putative ATP-dependent RNA helicase DHX30 | 15 | 4 | 3.35 | Jourdan et al. 2012. |
| RPS13 | 40S ribosomal protein S13 | 14 | 6 | 39.07 |  |
| MYO1C | Unconventional myosin-Ic | 14 | 6 | 17.52 |  |
| CPSF7 | Cleavage and polyadenylation specificity factor subunit 7 | 13 | 7 | 22.46 |  |
| EIF4A1 | Eukaryotic initiation factor 4A-I | 13 | 6 | 19.21 | Jourdan et al. 2012. |
| RPS3A | 40S ribosomal protein S3a | 13 | 5 | 30.40 |  |
| RPS18 | 40S ribosomal protein S18 | 13 | 4 | 21.71 |  |
| SET | N-lysine methyltransferase SETD8 | 13 | 3 | 10.19 |  |
| PARP1 | Poly [ADP-ribose] polymerase 1 | 13 | 2 | 2.76 |  |
| RPS9 | Submitted name: RPS9 protein | 12 | 4 | 20.14 |  |
| HNRNPA3 | Heterogeneous nuclear ribonucleoprotein A3 | 12 | 2 | 13.04 | Jourdan et al. 2012. |
| RPL38 | 60S ribosomal protein L38 | 12 | 2 | 34.29 |  |
| SLC25A11 | Submitted name: SLC25A11 protein | 12 | 6 | 23.57 |  |
| KRT1 | Keratin, type II cytoskeletal 1 | 12 | 2 | 3.73 |  |
| LRPAP1 | Alpha-2-macroglobulin receptor-associated protein | 12 | 5 | 17.09 |  |
| EIF3C | Eukaryotic translation initiation factor 3 subunit C | 12 | 6 | 9.93 | Jourdan et al. 2012. |
| C14orf166 | UPF0568 protein C14orf166 | 12 | 6 | 32.79 |  |
| NIPSNAP3A | Protein NipSnap homolog 3A | 11 | 4 | 19.43 |  |
| C1QBP | Complement component 1 Q subcomponent-binding protein, mitochondrial | 11 | 3 | 21.63 |  |
| RPS19 | 40S ribosomal protein S19 | 10 | 5 | 31.72 |  |
| CFL1 | Cofilin-1 | 10 | 4 | 40.36 |  |
| FADS2 | Fatty acid desaturase 2 | 10 | 3 | 8.56 |  |
| VDAC2 | Voltage-dependent anion-selective channel protein 2 | 9 | 2 | 12.55 |  |
| RPS5 | 40S ribosomal protein S5 | 9 | 3 | 14.22 |  |
| RPL22L1 | 60S ribosomal protein L22-like 1 | 9 | 2 | 19.83 |  |
| ZNF703 | Zinc finger protein 703 | 9 | 5 | 20.68 |  |
| MALT1 | Mucosa-associated lymphoid tissue lymphoma translocation protein 1 | 9 | 4 | 7.04 |  |
| HNRNPA1 | Heterogeneous nuclear ribonucleoprotein A1 | 8 | 4 | 19.62 | Jourdan et al. 2012. |
| AAMP | Angio-associated migratory cell protein | 8 | 2 | 8.92 |  |
| VPS41 | Vacuolar protein sorting-associated protein 41 homolog | 8 | 2 | 2.17 |  |
| RPS20 | 40S ribosomal protein S20 | 7 | 3 | 22.69 |  |
| RPS17L | 40S ribosomal protein S17-like | 7 | 2 | 15.56 |  |
| G3BP1 | Ras GTPase-activating protein-binding protein 1 | 7 | 2 | 8.10 |  |
| THUMPD3 | THUMP domain-containing protein 3 | 7 | 2 | 5.33 |  |
| UPF1 | Regulator of nonsense transcripts 1 | 6 | 5 | 5.31 | Jourdan et al. 2012. |
| CTBP1 | C-terminal-binding protein 1 | 6 | 5 | 13.29 |  |
| LARP1 | La-related protein 1 | 6 | 3 | 2.83 | Jourdan et al. 2012. |
| RPL10 | 60S ribosomal protein L10 | 6 | 2 | 8.41 |  |
| NUDT19 | Nucleoside diphosphate-linked moiety X motif 19, mitochondrial | 6 | 3 | 8.80 |  |
| SLC12A2 | Submitted name:  SLC12A2 protein | 6 | 9 | 12.71 |  |
| POLDIP3 | Submitted name:  Polymerase delta-interacting protein 3 | 6 | 2 | 13.10 |  |
| PSPH | Phosphoserine phosphatase | 6 | 4 | 20.86 |  |
| FARS2 | Phenylalanine--tRNA ligase, mitochondrial | 5 | 2 | 5.54 |  |
| RPS16 | 40S ribosomal protein S16 | 5 | 5 | 31.51 |  |
| CPNE3 | Submitted name: CPNE3 protein | 5 | 3 | 5.96 |  |
| RPS14 | 40S ribosomal protein S14 | 5 | 2 | 18.54 |  |
| TLE4 | Transducin-like enhancer protein 4 | 5 | 2 | 18.78 |  |
| SBDS | Ribosome maturation protein SBDS | 5 | 2 | 12.80 |  |
| SUCLG1 | Succinyl-CoA ligase [ADP/GDP-forming] subunit alpha, mitochondrial | 5 | 5 | 17.34 |  |
| TPR | DnaJ homolog subfamily C member 7 | 5 | 17 | 10.62 |  |
| AMMECR1 | AMME syndrome candidate gene 1 protein | 5 | 3 | 23.81 |  |
| RTCA | RNA 3'-terminal phosphate cyclase | 5 | 2 | 5.46 |  |
| KRT10 | Keratin, type I cytoskeletal 10 | 5 | 2 | 3.77 |  |
| LETM1 | LETM1 and EF-hand domain-containing protein 1, mitochondrial | 5 | 2 | 4.19 |  |
| CTTNBP2 | CTTNBP2 N-terminal-like protein  CTTNBP2N | 4 | 2 | 2.04 |  |
| MRPL14 | 39S ribosomal protein L14, mitochondrial | 4 | 3 | 24.83 |  |
| PUM2 | Submitted name: PUM2 protein. Drosophila version termed Pumilio homolog 2 | 4 | 2 | 2.68 | PUM1 identified in Jourdan et al. 2012. |
| INPP4A | Type I inositol 3,4-bisphosphate 4-phosphatase | 4 | 2 | 3.09 |  |
| MAPRE2 | Microtubule-associated protein RP/EB family member 2 | 4 | 8 | 49.27 |  |
| PRPF38A | Pre-mRNA-splicing factor 38A | 4 | 2 | 8.97 |  |
| SMAD5 | Mothers against decapentaplegic homolog 5 | 4 | 5 | 10.78 |  |
| INPP1 | Submitted name: INPP1 protein | 4 | 2 | 8.27 |  |
| RBM12B | Putative uncharacterized protein encoded by RBM12B-AS1 | 4 | 2 | 3.06 |  |
| EIF3A | Eukaryotic translation initiation factor 3 subunit A | 4 | 2 | 3.71 |  |
| ANAPC1 | Anaphase-promoting complex subunit 1 | 4 | 2 | 1.65 |  |
| RPS9 | Not assigned | 4 | 4 | 22.09 |  |
| HNRNPM | Heterogeneous nuclear ribonucleoprotein M | 4 | 2 | 5.37 |  |
| TAOK1 | Serine/threonine-protein kinase TAO1 | 4 | 2 | 2.80 |  |
| HNRNPA2/B1 | Not assigned | 4 | 3 | 12.75 | Jourdan et al. 2012. |
| EIF4A3 | Eukaryotic initiation factor 4A-III | 4 | 4 | 12.65 |  |
| CHID1 | Chitinase domain-containing protein | 4 | 4 | 18.07 |  |
| UQCR10 | Cytochrome b-c1 complex subunit 9 | 4 | 2 | 31.75 |  |

Jourdan et al. 2012. An interactome map of the nucleocapsid protein from a highly pathogenic North American porcine reproductive and respiratory syndrome virus strain generated using SILAC-based quantitative proteomics. Proteomics 12:1015-1023.

Wang et al. 2014. Poly(A)-binding protein interacts with the nucleocapsid protein of porcine reproductive and respiratory syndrome virus and participates in viral replication. Antiviral Research 96:315-323.
